# Preferential Infiltration of Unique Vγ9Jγ2-Vδ2 T Cells Into Glioblastoma Multiforme

**DOI:** 10.3389/fimmu.2019.00555

**Published:** 2019-03-22

**Authors:** Mijeong Lee, Chanho Park, Jeongmin Woo, Jinho Kim, Inseong Kho, Do-Hyun Nam, Woong-Yang Park, Yeon-Soo Kim, Doo-Sik Kong, Hye Won Lee, Tae Jin Kim

**Affiliations:** ^1^Department of Health Science and Technology, Samsung Advanced Institute for Health Science and Technology, Sungkyunkwan University, Seoul, South Korea; ^2^Institute for Refractory Cancer Research, Samsung Medical Center, Seoul, South Korea; ^3^Division of Immunobiology, Department of Molecular Cell Biology, Sungkyunkwan University School of Medicine, Suwon, South Korea; ^4^Samsung Genome Institute, Samsung Medical Center, Seoul, South Korea; ^5^Department of Neurosurgery, Samsung Medical Center, Sungkyunkwan University School of Medicine, Seoul, South Korea; ^6^Department of New Drug Discovery and Development, Chungnam National University, Daejeon, South Korea; ^7^Department of Anatomy and Cell Biology, Sungkyunkwan University School of Medicine, Suwon, South Korea; ^8^Single Cell Network Research Center, Sungkyunkwan University, Seoul, South Korea

**Keywords:** glioblastoma multiforme, tumor immune microenvironment, gamma-delta T cells, gamma-delta T-cell receptor repertoire, immune repertoire sequencing

## Abstract

Glioblastoma multiforme (GBM) is clinically highly aggressive as a result of evolutionary dynamics induced by cross-talk between cancer cells and a heterogeneous group of immune cells in tumor microenvironment. The brain harbors limited numbers of immune cells with few lymphocytes and macrophages; thus, innate-like lymphocytes, such as γδ T cells, have important roles in antitumor immunity. Here, we characterized GBM-infiltrating γδ T cells, which may have roles in regulating the GBM tumor microenvironment and cancer cell gene expression. V(D)J repertoires of tumor-infiltrating and blood-circulating γδ T cells from four patients were analyzed by next-generation sequencing-based T-cell receptor (TCR) sequencing in addition to mutation and immune profiles in four GBM cases. In all tumor tissues, abundant innate and effector/memory lymphocytes were detected, accompanied by large numbers of tumor-associated macrophages and closely located tumor-infiltrating γδ T cells, which appear to have anti-tumor activity. The immune-related gene expression analysis using the TCGA database showed that the signature gene expression extent of γδ T cells were more associated with those of cytotoxic T and Th1 cells and M1 macrophages than those of Th2 cells and M2 macrophages. Although the most abundant γδ T cells were Vγ9Vδ2 T cells in both tumor tissues and blood, the repertoire of intratumoral Vγ9Vδ2 T cells was distinct from that of peripheral blood Vγ9Vδ2 T cells and was dominated by Vγ9Jγ2 sequences, not by canonical Vγ9JγP sequences that are mostly commonly found in blood γδ T cells. Collectively, unique GBM-specific TCR clonotypes were identified by comparing TCR repertoires of peripheral blood and intra-tumoral γδ T cells. These findings will be helpful for the elucidation of tumor-specific antigens and development of anticancer immunotherapies using tumor-infiltrating γδ T cells.

## Introduction

Glioblastoma multiforme (GBM) progresses by co-opting stromal cells that reside in or are recruited to the tumor microenvironment (TME), which is a complex ecosystem composed of heterogeneous tumor cells associated with extensive hypoxic zones, reactive astrocytes, and infiltrating distinct immune components, including microglia, tumor-associated macrophages (TAMs) derived from peripheral blood monocytes, granulocytes, myeloid-derived suppressor cells (MDSCs), and T cells (1–5). In particular, GBM tumor cells along with immune factors create a complex milieu, which ultimately leads to alteration of the tumor cell transcriptome and tumor evolution (6, 7). TAMs, the predominant immune population infiltrating GBMs (1, 8), orchestrates GBM evolution by facilitating the mesenchymal transition, neoangiogenesis, extra-cellular matrix remodeling, and immune modulation (6, 9–11). Furthermore, accumulation of CD4^+^ T helper (Th) cells and CD4^+^CD25^+^ transcription factor forkhead box P3 (FoxP3)^+^ regulatory T cells (Tregs) combined with the reduced presence of CD8^+^ cytotoxic T cells results in curtailment of immunotherapeutic efficacy (9, 12).

However, the nature of GBM-infiltrating γδ T cells has not been extensively investigated, although γδ T cells have the potential to kill cancer cells and to change the pro-tumoral TME to one favoring acute responses and potent anti-tumoral activity. (13–19). The human TCR variable (V) regions of TCR γ and δ genes contains 14 unique Vγ segments (TRGV), three unique Vδ segments (TRDV1, TRDV2, and TRDV3), and five Vδ segments that share a common nomenclature with Vα segments (TRDV4/TRAV14, TRDV5/TRAV29, TRDV6/TRAV23, TRDV7/TRAV36, and TRDV8/TRAV38-2) (20). In healthy human adults, circulating T lymphocytes include 1–3% γδ T cells, most of which are Vγ9Vδ2 T cells (16). Vγ9Vδ2 T cells are activated by pyrophosphate-containing metabolites, generically known as phosphoantigens (pAgs), which are derived from microbes or metabolically active tumor cells and bind to the intracellular domain of the butyrophilin-related molecule BTN3A1 without major histocompatibility complex (MHC)- or CD1-dependent antigen presentations (18, 21). Especially, isopentenyl pyrophosphate (IPP) is a pAg generated from the mevalonate pathway in mammalian cells (22), and Vγ9Vδ2 T cells have additional sensor for detecting cancer cells via recognition of IPP accumulated intracellularly during dysregulated metabolism in cancer cells (16–18, 23). GBM cancer cells also express several MHC-like stress-induced self-antigens (MIC-A/B), heat shock protein-60, U16-binding protein 4, human MutS homolog 2, and F1-ATP synthase, which are recognized by TCRs, Toll-like receptors, or natural killer (NK) receptors expressed on Vγ9Vδ2 T cells, triggering cancer cell killing without any prior antigen exposure or priming (15, 24–26). Although the γδ T cell proliferative function is impaired in patients with GBM, *ex vivo*-expanded/activated γδ T cells from healthy donors are highly cytotoxic to GBM tumor cells (14), suggesting a therapeutic effects of the adoptive transfer of Vγ9Vδ2 T-cells as an alternative immunotherapeutic strategy GBMs (13, 19). For example, temozolomide (TMZ)-induced DNA damage upregulates NKG2D ligands on cancer cells that are vulnerable to Vγ9Vδ2 T cell-mediated lysis in GBM (27).

Immune repertoire sequencing (IR-SEQ) can offer a comprehensive snapshot of the complexity and the diversity of the TCR repertoire (28, 29). There are many advantages of studying the TCRγδ repertoire compared with repertoires of TCRαβ and B-cell receptors because of relatively limited diversity of γδ T cells and their independence from different MHC haplotypes (30). In the blood, the repertoire of Vγ9Vδ2 T cells is skewed toward cells expressing a biased Vγ9 chain with Vγ9-JγP-C1 rearrangement, which involves Vγ9 gene segment, JγP (Jγ1.2) joining segment, and Cγ1 exon (31, 32). The Vγ9-JγP-C1 chain is paired mainly with Vδ2 chains (31). Despite using common V-(D)-J rearrangements, circulating Vγ9Vδ2 cells are still diverse due to the existence of D fragments, N-addition occurring during V-(D)-J recombination, and alternative rearrangement with the Cγ2 segment (33, 34). In this study, we analyzed GBM-infiltrating γδ T cells with unique repertoire diversity although γδ T cells may be important innate lymphocytes modulating GBM TME. To evaluate the identity of central nervous system (CNS)-resident γδ T cells and determine whether blood γδ T cells were recruited to the tumor or whether local CNS-resident γδ T cells responded to the tumor, we performed γδ TCR repertoire analyses using tumor tissue and matched peripheral blood from four patients with GBM based on IR-SEQ technology.

## Materials and Methods

### Sample Collection and Preparation for Repertoire Sequencing

We recruited four cohorts of patients diagnosed with GBM. All patients signed informed consent for the use of patients' samples for research purposes under protocols approved by the Samsung Medical Center Institutional Review Board (IRB no. 2016-11-073). Tumor tissues were stored in liquid nitrogen, and peripheral blood mononuclear cells (PBMCs) were isolated from whole blood with a Ficoll Histoplaque gradient. PBMCs were labeled with fluorescein isothiocyanate-conjugated anti-CD45 antibodies (347463; BD Biosciences, San Jose, CA, USA), and CD45^+^ cells were acquired using a BD FACS AriaIII flow cytometer with FACSDiva software (BD Biosciences).

### Panel Sequencing Data Analysis

Samples were profiled using GliomaSCAN, a sequencing platform designed to target 312 genes specific for GBM, at the Samsung Medical Center. These target genes were chosen by literature mining or requested by the researchers and clinicians. The paired-end reads were aligned to the human reference genome (hg19) using Burrows-Wheeler Alignment tool (version 0.7.5). We converted sequence alignment and mapping (SAM) files into binary alignment and mapping (BAM) files using SAMtools (version 0.1.19) followed by reads sorting. Duplicated reads were removed from BAM files with Picard (version 1.128; http://broadinstitute.github.io/picard). Local realignment of reads around potential small indels and base quality score recalibration was performed with the Genome Analysis Toolkit (GATK, version 3.5.0) using dbSNP (build ID 137). Single nucleotide variants and indels were called using muTect2 (GATK version 3.8.0) and Strelka2 (version 2.8.2) with default parameter settings. The union of the variants identified by the two callers was used as the candidate variants. Variants were annotated using ANNOVAR. Variants located in exonic regions with a variant allele frequency of ≥ 0.1 were chosen for further investigation.

### Whole-Transcriptome Sequencing (WTS) and Enumeration of Immune Cell Subsets From WTS

Total RNA from human tissue was isolated with an RNeasy mini kit (#74106; Qiagen, Hilden, Germany). The biospecimens used for this study were provided by Samsung Medical Center BioBank (IRB no. 2016-11-073). For all samples, RNA-Seq libraries were prepared from 500 ng total RNA using an Illumina TruSeq RNA Sample Prep kit. All libraries were sequenced to a read depth of more than 75 million reads using an Illumina HiSeq2000 instrument to generate paired reads ends with a total read length of 100 bp. After trimming poor-quality reads and adapter sequences from the FASTQ files for each sample, we aligned the reads to the human reference genome (hg19) using STAR (version 2.5.0i) with two pass default mapping mode (35). With the same reference genome used for mapping, gene annotation data obtained from Ensemble (v 74) were used to quantify aligned reads. Transcripts per million normalized values for each gene were calculated based on total gene read counts and the lengths of merged exons using RSEM (version 1.2.17) (36). *In silico* deconvolution analysis was performed with transcriptomic data using the CIBERSORT algorithm under the default mode (37). The proportions of 22 immune cell types, including seven T-cell types, naïve and memory B cells, plasma cells, NK cells, and myeloid subsets were estimated using LM 22 datasets, which included the public gene signature matrix of 547 genes to distinguish 22 leukocyte subsets.

### Immune Cell Signature Analysis

Using curated immune gene expression signature (as shown in Supplementary Table 1) (38–41), gene set variation analysis (GSVA) was implemented to calculate sample wise enrichment scores for each immune related gene set using the Bioconductor package ‘GSVA’ (42) based on the TMM normalized WTS data from four GBM samples used in this study and TCGA-GBM dataset. GSVA scores were scaled and plotted using heatmap.2 function from *ggplot2* (43). RPKM normalized RNA-seq datasets for 170 samples from TCGA were used for GSVA analysis. Interrelations of all possible pairs of GSVA scores of Immune signature and gene expression values of γδ T cell related genes were estimated from Pearson's correlation coefficient (*r*). To infer significance of each correlation, *P*-values were calculated based on asymptotic t approximation. Hierarchical clustering of immune signature gene sets (column) and γδ T cell related gene expressions (row) was performed via Pearson's correlation to measure distance with complete linkage algorithm for clustering distances.

### Library Preparation and Sequencing

The human TCRγ and TCRδ CDR3 regions were amplified using the commercially available iRepertoire platform (iRepertoire Inc., Huntsville, AL, USA) in LAS Inc. (Kimpo, Gyeonggi-do, Korea). Briefly, total RNA samples were subjected to reverse transcription polymerase chain reaction (PCR) using iR-PCR1 Rxn Mix (iRepertoire Inc.) and PCR1 Rxn Mix (iRepertoire Inc.). The PCR product was purified using PCR1 Rescue Mix (iRepertoire Inc.). A second PCR was then carried out using PCR2 Mix again and the product was purified using PCR2 Clean-up Mix (iRepertoire Inc.). Finally, quality and band size of libraries were assessed using an Agilent 2100 bioanalyzer (Agilent, Santa Clara, CA, USA). Libraries were quantified by quantitative PCR using CFX96 Real Time System (Bio-Rad, Hercules, CA, USA). After normalization, sequencing of the prepared library was conducted on the Miseq system (Illumina, San Diego, CA, USA) with 250-bp paired-end reads.

### Analysis of the TCR Repertoire With High-Throughput Sequencing

Using an Illumina MiSeq system, we obtained 250-bp paired-end reads files, and the raw paired-end fastq files were analyzed using the Immune Repertoire High-throughput Sequence Analysis (IRSA) workflow at the iRepertoire website (https://irweb.irepertoire.com/nir/). The IRSA workflow included storing and managing sequencing data, removing sequencing artifacts, mapping reference sequences using the Smith-Waterman algorithm, identifying CDR3 junctions, and generating various distribution plots, such as domain usage, nucleotide nibbling, addition at the junction sites, and CDR3 length. Contigs of TCR constant genes were screened within quality control-filtered RNA-Seq data using the K-mer search algorithm of BBDuk program from the BBMap 35.74 suite (http://sourceforge.net/projects/bbmap/) with the parameter “k = 25 edist = 2”. Constant genes for α-, β-, γ-, and δ-type TCRs used for the query were obtained from IMGT/LIGM-DB version 1.2.4 (44). Accession code X02883 was used to retrieve the *TRAC* gene of TCRα; M12887 and L36092 for exons 1 and 2 of *TRBC1*; M12888 and L36092 for two exons of the *TRBC2* gene of TCRβ; M14996, M14997, and M14998 for three exons of *TRGC1*; M14002 for *TRGC2* of TCRγ; and M22149, M22150, and M22151 for three exons of *TRDC1* of TCRδ. Additionally, TCR Repertoire Utilities for Solid Tissue (45) was used to detect TCR sequences from RNA-Seq data for individual samples.

### Immunohistochemistry (IHC)

IHC staining was performed using OpalTM 7-color manual kit (NEL81100KT, PerkinElmer, MA, USA) according to the manufacturer's protocol (2014;70:46-58). Briefly, the slides were deparaffinized in xylene and rehydrated in ethanol. Antigen retrieval was performed in tris-buffered saline buffer (pH 9.0) using microwave treatment (MWT). Using two antibodies are listed as follow: TCR gamma/delta antibody (2 μg/mL, mouse monoclonal, (5A6.E9), TCR1061, Thermofisher, MA, USA) and CD204 (1 μg/ml, rabbit polyclonal, ab64693, abcam, Cambridge, UK). These two antibodies were incubated 30 min in a humidified chamber at room temperature, followed by detection using the mouse/rabbit SuperPicture Polymer Detection HRP kit. Visualization of the primary antibody was accomplished using each Opal Fluorophore Working Solution (TSA, 1:100), after which the slide was placed in tris-buffered saline buffer (pH 9.0) and repeated using MWT. TCR gamma/delta and CD204 were visualized with opal 690 and 520, respectively. Nuclei were subsequently visualized with DAPI and the slide was coverslipped using the antifade mounting solution (ADI-950-260-0025, Enzo, NY, USA). The slides were examined with Vectra Polaris Automated Quantitative Pathology Imaging System (PerkinElmer). InForm image analysis software (PerkinElmer) was used to analyze the spectra for all fluorophores included from 420 to 720 nm.

### Availability of Data and Material

Newly generated GliomaSCAN, WTS, and γδ TCR repertoire-Seq data from this study can be accessed at the European Genome-phenome Archive with accession number EGAS00001002790.

## Results

### Clinical Presentation of Four Patients With Isocitrate Dehydrogenase (IDH) 1 Wild-Type GBM

**Figure 1:**
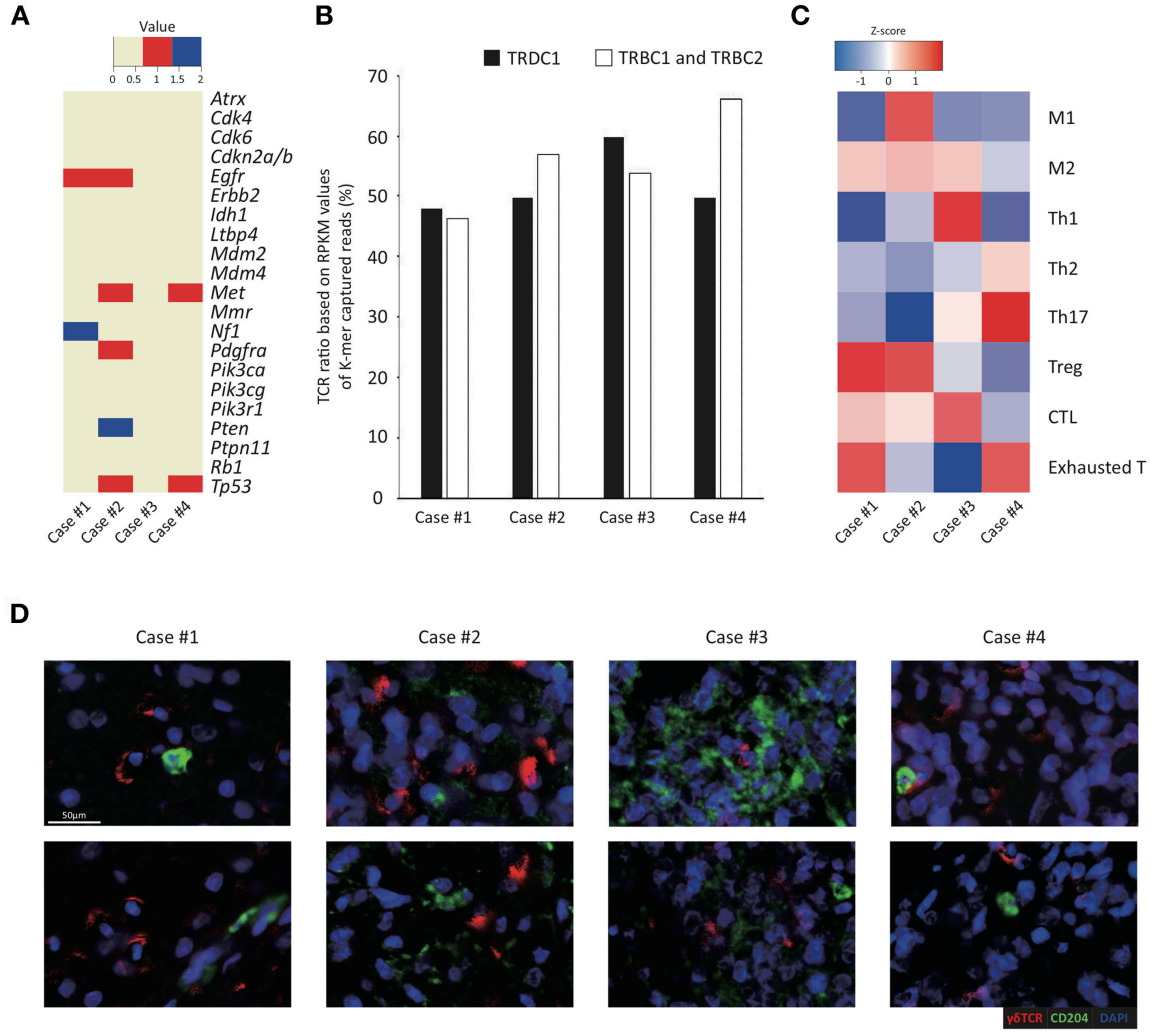
Tumor-infiltrating γδ T cells and immune profiles in four GBM cases. **(A)** Summary of mutational changes in 21 GBM-related genes across sequenced samples. Red and blue denote single nucleotide variant events and indel events, respectively. **(B)** RPKM values (y-axis) of TCRB and TCRD captured reads from RNA-seq data of four cases (x-axis) are shown as a column chart. **(C)** The heatmap illustrates the z-score of normalized Gene Set Variable Analysis (GSVA) enrichment scores of a variety of curated immune gene signatures based on whole transcriptome sequencing data of four GBM samples analyzed in this study. The z-scores are calculated based on average and standard deviation of GSVA enrichment scores of each patient, indicating relative enrichment scores of immune gene sets within each patient. The scale of z-score is shown: red illustrates high enrichment and blue illustrates low enrichment. **(D)** The representative images of the multiplex immunohistochemistry analysis of four GBM patient tissue samples stained for γδ TCR in red and CD204 in green. Scale bar, 50 μm.

The patients' clinical course and therapeutic protocols are summarized in Supplementary Figure 1 and Table 1. Confirmation of IDH1 wild-type, O^6^-methylguanine-DNA-methyltransferase (MGMT) promoter-unmethylated GBM was made using R132 sequencing and methylation-specific PCR according to the 2016 World Health Organization criteria (46) after gross total resection (GTR). Four cases of GBM showed different genetic mutations with no mutation in the *IDH1* gene (Figure 1A). The first patient (case 1) was a 52-year-old woman with a large abnormal enhanced mass on the right temporal lobe on brain magnetic resonance imaging (MRI). She received concurrent chemoradiotherapy with TMZ followed by adjuvant TMZ (localized brain radiotherapy, total 60 Gy in 2 Gy per daily fraction with daily 75 mg/m^2^ TMZ over 6 weeks followed by adjuvant cycles of TMZ 150 mg/m^2^/day for 5 days during each 28-day cycle) (47), and brain MRI during four adjuvant TMZ courses revealed tumor recurrence around the resection site. For chemotherapy against recurrent GBM, she was treated with bevacizumab 10 mg/kg and irinotecan 125 mg/m^2^. The second patient (case 2) was a 70-year-old man suffering from left temporo-occipital GBM with MGMT promoter-unmethylation. The GTR of the primary tumor was confirmed on the post-operative follow-up brain MRI. After concurrent chemoradiation and two adjuvant TMZ courses, newly developed multifocal nodular enhancing lesions at the subependymal area of both lateral ventricles suggesting leptomeningeal seeding were found. Bevacizumab 10 mg/kg and irinotecan 125 mg/m were administered. The third patient (case 3) was a 60-year-old woman with a right temporal GBM with MGMT promoter-methylation. After GTR of the primary tumor was performed, she was treated with concurrent chemoradiotherapy with TMZ followed by adjuvant TMZ with no obvious disease relapse during the follow-up period of 10 months. Final fourth patient (case 4) had a left temporo-parietal GBM with MGMT promoter-methylation. She received standard concurrent chemoradiation plus adjuvant TMZ with no obvious disease relapse during the follow-up period of 14 months.

**Table 1:** Clinical characteristics of four glioblastoma patients.

**Case**	**Age**	**Gender**	**Tumor location**	**MGMT promoter methylation status**	**Post-adjuvant treatment**	**PFS (Days)**	**OS (Days) (all censored)**
#1	52	Female	Rt. T	Un-methylated	Radiotherapy plus concomitant and adjuvant temozolomide	207 (progression during F/U)	270
#2	70	Male	Lt. T-O	Un-methylated	Radiotherapy plus concomitant and adjuvant temozolomide (#2)	144 (progression during F/U)	173
					+ Bevacizumab plus irinotecan after disease recurrence		
#3	60	Female	Rt. T	Methylated	Radiotherapy plus concomitant and adjuvant temozolomide	198 (Censored, no progression during F/U)	198
#4	61	Female	Lt. T-P	Methylated	Radiotherapy plus concomitant and adjuvant temozolomide	249 (Censored, no progression during F/U)	249

*Lt., left; Rt., right; T-P, temporo-parietal; T, temporal; T-O, temporo-occipital; GTR, gross total resection; IDH1, Isocitrate dehydrogenase 1; WT, wild type; MGMT, O6-methylguanine-DNA-methyltransferase; RT, radiotherapy; TMZ, temozolomide; PFS, progression-free survival; OS, overall survival; F/U, follow-up*.

### Abundant Infiltration of Macrophages and Innate and Effector/Memory Lymphocytes in GBM Tumor Tissues

To estimate antitumor immunity in GBM pathogenesis, we obtained gene expression profiles of four GBM tissues and measured the relative proportions of various immune cells through *in silico* deconvolution analysis **(**CIBERSORT; see Supplementary Figure 2). In all four cases, heavy infiltration of macrophages (32.0–80.3% of all immune cells), particularly M2 macrophages, and mast cells (6.7–34.6% of all immune cells) was noted. T and NK cells constituted about 5.6–31.0% of all immune cells, and most tumor-infiltrating lymphocytes (TILs) were innate and effector/memory cells, whereas naïve T cells were scarce. However, the enumeration of tumor-infiltrating γδ T cells via the CIBERSORT algorithm was difficult since γδ T cells also express most genes expressed in αβ T cells and NK cells (37, 48). Alternatively, the ratios of γδ T to αβ T cells were calculated to be around 0.8–1.1 by measuring the reads per kilobase of transcript per million mapped reads (RPKM) values of the *Tcrd* and *Tcrb* constant regions, suggesting that the ratios of γδ T cells were equal to or even higher than those of αβ T cells in the tumor tissues (Figure 1B). The case 3 showed the highest ratio of γδ T cells than the other cases, whereas the case 4 showed a lower ratio of γδ T cells than other cases.

To explore the effects of γδ T cell infiltration, the phenotype of immune cells within the TME in four GBM samples was then analyzed based on multiple gene sets curated from various sources (summarized in Supplementary Table 1). In all four cases, heterogeneous and complex activation status of specific tumor-associated immune subsets modulating pro- or anti-tumor activity was demonstrated in each case. Notably, a relatively high abundance of cytotoxic T cell and Th1 genes was detected in case 3, which showed the highest ratio of γδ T cells over αβ T cells (Figure 1C). In contrast, case 4 tumor samples highly expressed Th17 and exhausted T cell genes compared to other samples. In case 1, genes related to M2 macrophage, Treg and exhausted T cells shaping immune-suppressive TME were relatively upregulated (Figure 1C). In further IHC study by using deparaffinized section slides, γδ T cells and TAMs were abundantly distributed throughout the tumor in all four cases and γδ T cells and macrophages were closely associated (Figure 1D).

**Figure 2:**
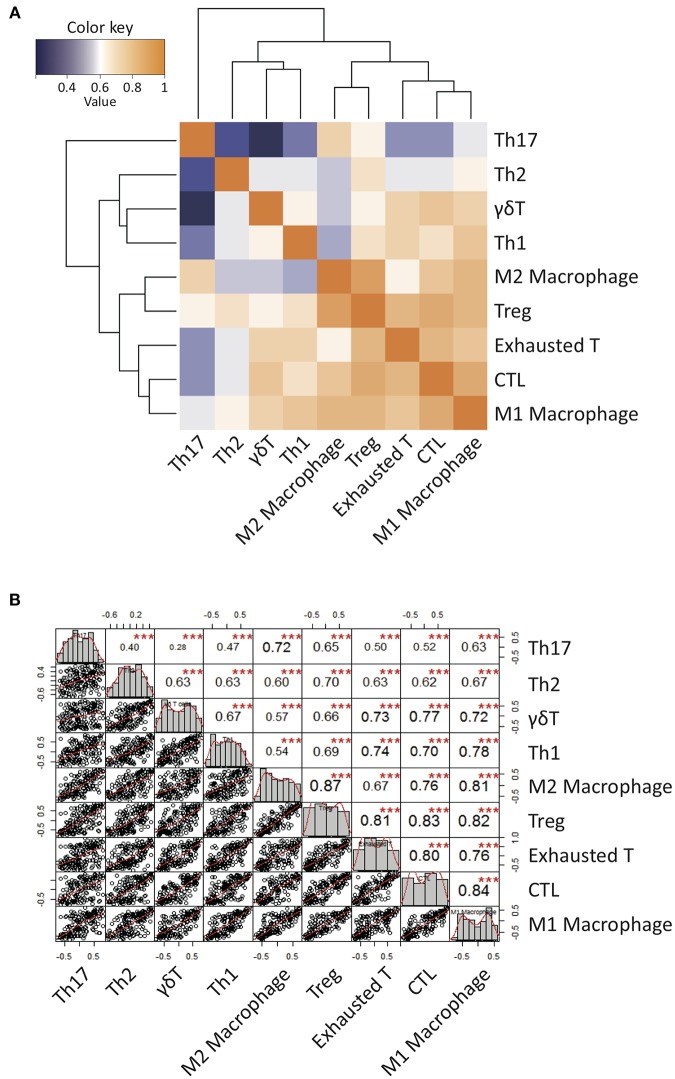
Correlation patterns of Gene Set Variable Analysis (GSVA) enrichment scores of a variety of curated immune gene signatures for 170 GBM patients from TCGA database. **(A)** A clustered heatmap of Pearson's correlation coefficient of GSVA scores over all immune signature gene sets. Hierarchical clustering was performed based on Pearson's correlation distance with ward linkage method. Dark orange denotes high correlation (*r* = 1) while dark blue denotes a lack of correlation (*r* > 0). **(B)** The diagonal of the chart represent distribution of GSVA enrichment scores. The bivariate scatter plots of linear regression fits of each pairs with the fitted lines are shown on the bottom of the diagonal. Pearson's correlation coefficient values with the level of significance were shown on the top of the diagonal. ^***^P < 0.001.

We presumed that the abundance of γδ T cells may be correlated with TME, but we could not draw clear conclusion due to small sample sizes. To overcome this limitation, we further examined patient populations from the published dataset from TCGA RNA-seq (*N* = 170) (Figure 2). Interestingly, the gene signature score of γδ T cells more positively correlated with those of M1 macrophages and Th1 cells than those of M2 macrophages and Th2 cells, respectively. Notably, the γδ T cell activity was strongly associated with cytotoxic T cell activity, suggesting that GBM-infiltrating γδ T cells contribute to anti-tumoral immunity. However, the γδ T cell activity was also associated with the activities of exhausted and regulatory T cells. These findings are consistent with previous findings demonstrating that γδ T cells show a high degree of plasticity and are able to assume different phenotypes, including Th1-like, Th2-like, Th17-like, follicular Th-like, or Treg-like characteristics, depending on the cytokine milieu in surrounding microenvironment (49–55). Based on the hypothesis that the γδ T cells could be responsible for the polarization of TAMs, we performed repertoire analyses of γδ T cells since individual subsets of γδ T cells with specific variable domains of γ or δ chains have unique functions in local environments.

### Predominant Infiltration of Vγ9Vδ2 T Cells in GBM Tumor Tissues

**Figure 3:**
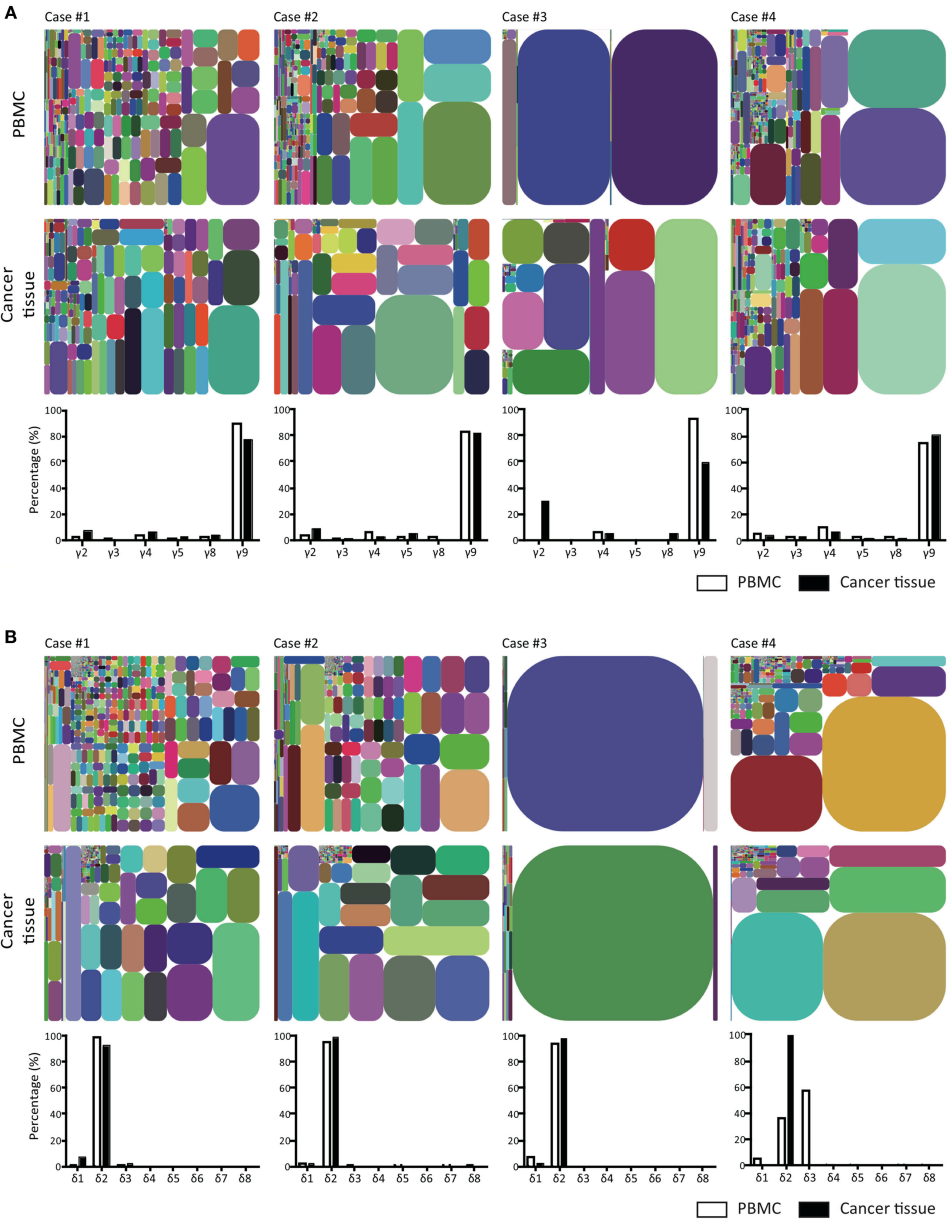
T cell receptor γ and δ chain CDR3 tree map plots for blood and GBM tumor tissue γδ T cells and the relative proportions of individual TCR TCR Vγ or Vδ gene segments. **(A)** for TCRγ **(B)** for TCRδ. CDR3 tree-map plots illustrate the unique TCRγ or TCRδ CDR3 nucleotide sequences obtained from given samples of four GBM patients. Each rectangle in a given tree-map represents a unique CDR3 sequence, and the size of each rectangle indicates the relative frequency of an individual sequence. The colors for the individual CDR3 sequences in each tree-map plot were chosen randomly. Bottom graphs in **(A,B)** show the percentages of use of each TCR Vγ **(A)** or Vδ **(B)** domain in PBMCs (white) or cancer tissues (black) from each patient.

To compare γδ TCR repertoires between tumor-infiltrating and blood γδ T cells and among γδ T cells from four patients, we generated and amplified *Tcrg* and *Tcrd* cDNA libraries from tumor tissues and sorted (2 × 10^5^ to 8 × 10^6^) blood CD3^+^ cells from four patients. The libraries, which could be distinguished by barcodes, were then pooled and sequenced (Illumina MiSeq). In total, 159,138–2,483,772 total clean nucleotide sequences (CNTs) and about 141–1,157 unique clean nucleotide sequences (CNUs) from the tumor samples and 402,922–2,145,312 CNTs and 93–2,709 unique CNUs from the sorted blood CD3^+^ cells were analyzed in this study (Table 2). The *TCRG* and *TCRD* tree maps for each γδ T cell population showed that intratumoral γδ T cells expressed less diverse CDR3 nucleotide sequences for the *TCRG* and *TCRD* genes than blood γδ T cells, except in case 3 (Figures 3A,B). Case 3 showed oligoclonal γδ T cells in both blood and tumor tissue. The calculation of Shannon indices, which reflected both richness and evenness of the CDR3 clonotypes, showed that the repertoires of *TCRG* and *TCRD* CDR3 sequences were very restricted in case 3 and that the intratumoral γδ T cells were less diverse than blood γδ T cells (Table 2). In all cases, the most abundant γδ T cells were of Vγ9 and Vδ2 cells.

**Table 2:** γδ T cell receptor repertoire diversity indices.

**Case #**	**TCR chain**	**Cell origin**	**Shannon index**	**Janssen-Shannon divergence**
#1	γ chain	PBMCs	6.841	0.738
		Cancer tissue	6.087	
	δ chain	PBMCs	7.599	0.802
		Cancer tissue	5.156	
#2	γ chain	PBMCs	5.665	0.543
		Cancer tissue	4.736	
	δ chain	PBMCs	6.069	0.560
		Cancer tissue	4.467	
#3	γ chain	PBMCs	1.458	1
		Cancer tissue	3.270	
	δ chain	PBMCs	0.624	0.999
		Cancer tissue	0.661	
#4	γ chain	PBMCs	5.301	0.795
		Cancer tissue	4.609	
	δ chain	PBMCs	3.612	0.771
		Cancer tissue	2.905	

### Unique Vγ9Jγ2-Vδ2 T Cells Were Distinctively Found in GBM Tumor Tissues, but Not in Blood

**Figure 4:**
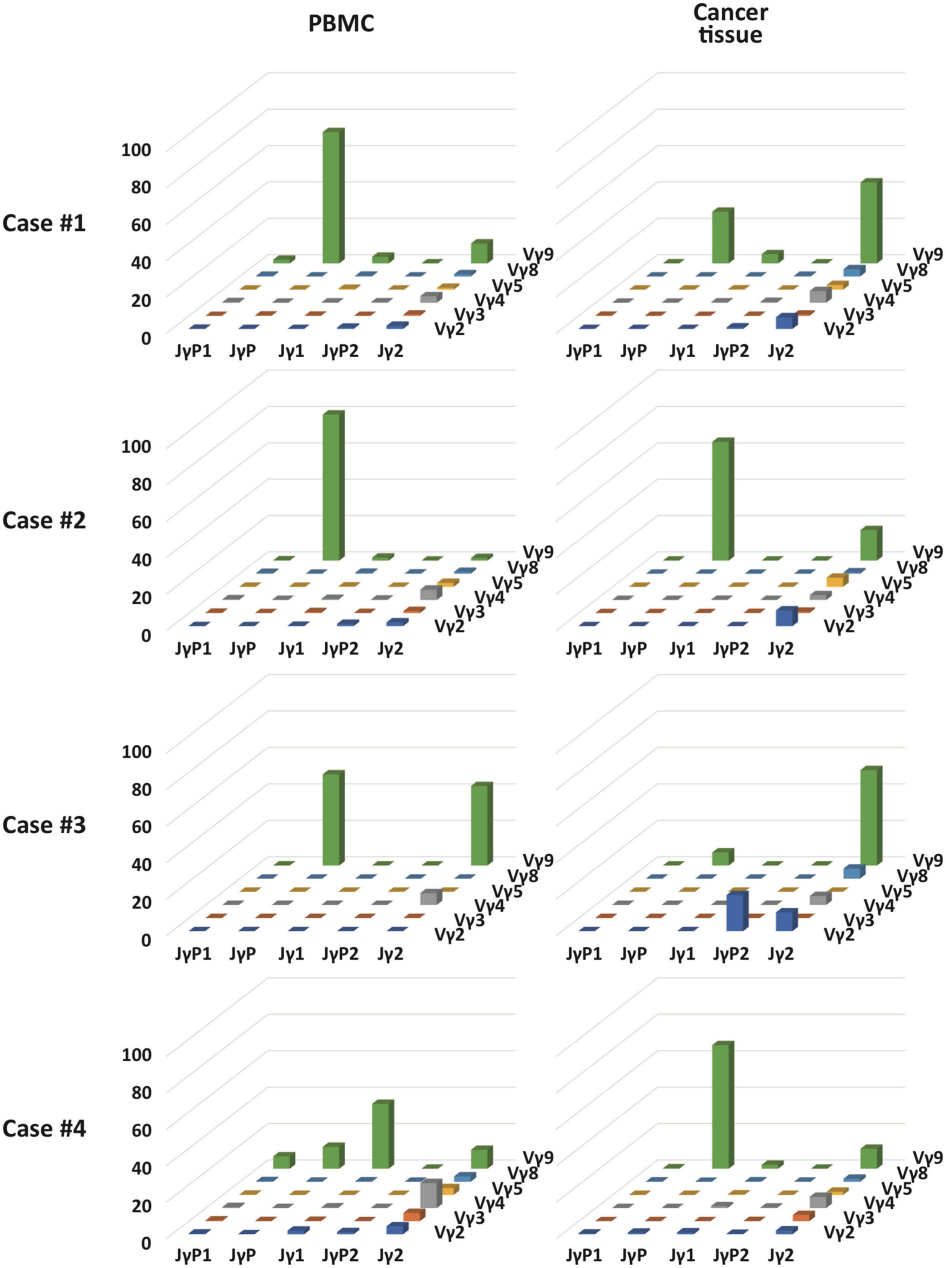
The relative proportion of each combination of Vγ and Jγ segments in γδ T cells in blood and GBM tumor tissue samples of four patients. 3D graphs show the relative percentages of combinations of Vγ-Jγ usages in PBMCs or GBM tumor tissues from four patients.

Vγ9Vδ2 T cells are known to be the most abundant type of adult blood γδ T cells (56). These cells undergo rapid expansion within the first year of life (57). Because Vγ9Vδ2 T cells are strongly activated by bacteria-derived pAgs (18, 21), these cells appear to be expanded by commensal bacteria (18). A few previous TCR repertoire analyses have revealed the predominance of canonical Vγ9JγP sequences (58). Although intratumoral and blood Vγ9Vδ2 T cells were apparently similar, the Jensen-Shannon divergence (JSD) indices, a measure of repertoire comparison (59), showed remarkable distinctness of intratumoral γδ T cells from blood γδ T cells (Table 2). Notably, in case 3, intratumoral γδ T cells were completely distinct from blood γδ T cells (JSD = 1 indicates complete divergence of two repertoires). When we plotted the clonotypes based on Vγ and Jγ segments, the absolute or relative abundance of Vγ9Jγ2 sequences was prominent in intratumoral γδ T cells, except in case 4 (Figure 4). The canonical Vγ9JγP sequences were the most abundant sequences in blood γδ T cells, except in case 4. Case 4 was unique in that many non-canonical Vγ9Jγ1, Vγ9Jγ2, Vγ9JγP1, and Vγ9JγP2 sequences as well as the canonical Vγ9JγP sequences were found in blood γδ T cells, as was verified through repetitive sequencing analysis. Paradoxically, the canonical Vγ9JγP sequences were the most abundant in intratumoral γδ T cells from case 4. Collectively, the non-canonical Vγ9Jγ2-Vδ2 T cells were distinctively observed in intratumoral γδ T cells in three of four 4 GBM cases.

### Unique CDR3 Vγ9Jγ2 and Vγ9JγP Clonotypes From GBM Tumor Tissues and Blood

To date, it is unclear whether there were variations in TCR Vγ9 γ chains related to antigenic specificity. In this study, we attempted to identify shared clonotypes that were found in different samples, although the antigenic specificity could not be deduced from the sequences. We reasoned that clonotypes of tumor phospho-antigen-specific Vγ9Vδ2 T cells may be different from blood bacterial phospho-antigen-specific Vγ9Vδ2 T cells. We listed the top 10 CDR3 clonotypes of canonical Vγ9JγP or non-canonical Vγ9Jγ2 sequences from four blood and tumor samples (Table 3). We found that the Vγ9JγP CDR3 clonotype ALWEVQELGKKIKV was the most frequent clonotype in blood and tumor samples from three cases, suggesting that this was the most representative clonotype of canonical Vγ9JγP γ chains. However, other frequent Vγ9JγP γ chain CDR3 clonotypes were also found, such as ALWEPPQELGKKIKV, in both blood and tumor samples from only case 1, ALWEKQELGKKIKV in the blood sample from case 2, and ALWEALRLGKKIKV in blood and tumor samples from case 3. Notably, we found several frequent Vγ9Jγ2 γ chain CDR3 clonotypes that were unique to tumors and were not found in blood. The representative Vγ9Jγ2 γ chain clonotypes included ALWEGLKKL in case 1, ALWEVQYKKL in case 2, ASKKTKKL in case 3, and ALWEVRYYYKKL in case 4. In case 2, ALWESSNYYKKL was a Vγ9Jγ2 γ chain clonotype found only in the blood. Collectively, we could discern unique blood- and tumor-specific Vγ9 γ chain CDR3 clonotypes and found that many tumor-specific clonotypes were of Vγ9Jγ2 γ chains, and many blood-specific clonotypes were of Vγ9JγP γ chains. In particular, the Vγ9JγP CDR3 clonotype ALWEVQELGKKIKV appeared to be the most representative Vγ9JγP γ chains. The γδ T cells with this clonotype appeared to be recruited to the tumor tissues from the blood.

**Table 3:** TOP 10 CDR3 sequence by the usage proportion of Vγ9Jγ2 or Vγ9JγP in patient blood and tissue sample.

**CASE #1**	**PBMCs**			**Tissue**		
**Vγ9 Jγ2**	**CDR3 amino acid sequence**	**% of Vγ9Jγ2 sequences**	**% of total sequences**	**CDR3 amino acid sequence**	**% of Vγ9Jγ2 sequences**	**% of total sequences**
1	ALWARLYYKKL	17.86	1.96	ALWEVRYYYKKL	27.19	12.09
2	ALWEVQPPYYKKL	5.47	0.60	ALWARLYYKKL	12.03	5.35
3	ALWEVSLYKKL	5.32	0.58	ALWECHYYKKL	6.81	3.03
4	ALWEVAPLYKKL	4.82	0.53	ALWEGNYYKKL	3.92	1.74
5	ALWESPYYKKL	4.35	0.48	ALWEAPKNTL	3.61	1.60
6	ALARKKL	4.31	0.47	ALWEGVNYYKKL	3.51	1.56
7	ALWEVLRYKKL	4.29	0.47	ALWEVQF	3.43	1.52
8	ALWDKKL	4.25	0.47	ALWEVLLEKL	3.24	1.44
9	ALWNKKL	4.20	0.46	ALWEVPYYKKL	2.86	1.27
10	ALWEVRAWVYYKKL	4.17	0.46	ALWVYKKL	2.85	1.27
**Vγ9 JγP**	**CDR3 amino acid sequence**	**% of Vγ9JγP sequences**	**% of total sequences**	**CDR3 amino acid sequence**	**% of Vγ9JγP sequences**	**% of total sequences**
1	ALWEVQELGKKIKV	17.76	12.83	ALWEVQELGKKIKV	5.14	5.14
2	ALWEVRELGKKIKV	5.31	3.83	ALWEVRELGKKIKV	3.83	3.83
3	ALWEVQRELGKKIKV	3.04	2.20	ALWEDQELGKKIKV	2.16	2.16
4	ALWEPLGELGKKIKV	2.75	1.98	ALWDKQELGKKIKV	1.57	1.57
5	ALWEELGKKIKV	2.71	1.96	ALWVVELGKKIKV	1.43	1.43
6	ALWEAQELGKKIKV	2.68	1.93	ALWEVMELGKKIKV	1.36	1.36
7	ALWEVQGLGKKIKV	2.49	1.80	ALWEPPELGKKIKV	1.35	1.35
8	ALWEEKELGKKIKV	2.30	1.66	ALWAVELGKKIKV	1.25	1.25
9	ALWDKQELGKKIKV	2.26	1.63	ALLGKKIKV	1.18	1.18
10	ALWEVRRELGKKIKV	2.21	1.59	ALWEVNQELGKKIKV	1.09	1.09
**CASE #2**	**PBMCs**			**Tissue**		
**Vγ9 Jγ2**	**CDR3 amino acid sequence**	**% of Vγ9Jγ2 sequences**	**% of total sequences**	**CDR3 amino acid sequence**	**% of Vγ9Jγ2 sequences**	**% of total sequences**
1	ALWEAPVEKL	26.04	0.37	ALWEGLKKL	17.58	2.95
2	ALSMQSFGYKKL	16.47	0.23	AFENYYKKL	17.07	2.87
3	ALWEGPKKL	16.19	0.23	ALEDKL	15.50	2.60
4	ALWEEDPDYKKL	13.80	0.19	ALWEVYV	15.35	2.58
5	ALWEVPAGYKKL	12.40	0.17	ALWEVLYYKKL	13.62	2.29
6	ALWEVRDYYKKL	6.69	0.09	ALSNYKKL	13.32	2.24
7	ALWRIYYKKL	4.15	0.06	ALWEFLYWGKL	5.38	0.90
8	ALGGGKL	3.60	0.05	ALEDKF	0.66	0.11
9	ALWEASVEKL	0.15	0.00	ALGDKL	0.47	0.08
10	ALGGGKF	0.13	0.00	ALEDRL	0.15	0.03
**Vγ9 JγP**	**CDR3 amino acid sequence**	**% of Vγ9JγP sequences**	**% of total sequences**	**CDR3 amino acid sequence**	**% of Vγ9JγP sequences**	**% of total sequences**
1	ALWEPPQELGKKIKV	22.95	18.42	ALWEPPQELGKKIKV	31.41	20.52
2	ALWEYRQELGKKIKV	8.59	6.89	ALWERELGKKIKV	9.49	6.20
3	ALWEVQELGKKIKV	8.34	6.70	ALWEVEELGKKIKV	8.13	5.31
4	ALWEAQELGKKIKV	7.64	6.13	ALWEVQELGKKIKV	7.65	4.99
5	ALWEVEELGKKIKV	6.15	4.94	ALWEPQELGKKIKV	6.75	4.41
6	ALWEPQELGKKIKV	5.70	4.58	ALWEDNSPKLGKKIKV	4.50	2.94
7	ALWELLEQELGKKIKV	4.91	3.94	ALWERQELGKKIKV	4.25	2.78
8	ALWEVPRELGKKIKV	3.68	2.95	ALWAQELGKKIKV	4.05	2.65
9	ALWEGEQELGKKIKV	2.88	2.32	ALWELLEQELGKKIKV	4.03	2.64
10	ALWEVQEELGKKIKV	2.50	2.01	ALWEGRELGKKIKV	4.03	2.63
**CASE #3**	**PBMCs**			**Tissue**		
**Vγ9 Jγ2**	**CDR3 amino acid sequence**	**% of Vγ9Jγ2 sequences**	**% of total sequences**	**CDR3 amino acid sequence**	**% of Vγ9Jγ2 sequences**	**% of total sequences**
1	ALWESSNYYKKL	98.22	42.79	ALWEVQYKKL	55.75	29.18
2	ALWEPSNYYKKL	0.44	0.19	ALWEHYYKKL	31.20	16.33
3	ALWGSSNYYKKL	0.33	0.14	ALWEVREKL	12.08	6.32
4	ALWESSSYYKKL	0.26	0.11	ALWGHYYKKL	0.21	0.11
5	ALWESPNYYKKL	0.21	0.09	ALWEVREEL	0.18	0.10
6	ALCESSNYYKKL	0.21	0.09	ALWEVQHKKL	0.10	0.05
7	ALWESSDYYKKL	0.15	0.07	ALWEYYYKKL	0.08	0.04
8	ALWKSSNYYKKL	0.07	0.03	ALWEHHYKKL	0.07	0.04
9	ALWVSSNYYKKL	0.04	0.02	ALWEVQCKKL	0.07	0.04
10	ALWESSYYYKKL	0.01	0.01	ALWEMQYKKL	0.04	0.02
**Vγ9 JγP**	**CDR3 amino acid sequence**	**% of Vγ9JγP sequences**	**% of total sequences**	**CDR3 amino acid sequence**	**% of Vγ9JγP sequences**	**% of total sequences**
1	ALWEKQELGKKIKV	98.65	49.31	ALWAQELGKKIKV	96.51	6.89
2	ALWEEQELGKKIKV	0.34	0.17	ALGAQELGKKIKV	2.17	0.15
3	ALWGKQELGKKIKV	0.26	0.13	ALWAQGLGKKIKV	0.37	0.03
4	ALWERQELGKKIKV	0.26	0.13	ALWARELGKKIKV	0.27	0.02
5	ALWEKRELGKKIKV	0.23	0.12	ALRAQELGKKIKV	0.24	0.02
6	ALWEKQKLGKKIKV	0.05	0.03	ALWTQELGKKIKV	0.21	0.02
7	ALWKKQELGKKIKV	0.05	0.03	ALWAQVLGKKIKV	0.08	0.01
8	ALWVKQELGKKIKV	0.03	0.01	ALWPQELGKKIKV	0.06	0.00
9	ALWEKHELGKKIKV	0.02	0.01	ALWAQKLGKKIKV	0.05	0.00
10	ALCEKQELGKKIKV	0.02	0.01	ALWAQEVGKKIKV	0.04	0.00
**CASE #4**	**PBMCs**			**Tissue**		
**Vγ9 Jγ2**	**CDR3 amino acid sequence**	**% of Vγ9Jγ2 sequences**	**% of total sequences**	**CDR3 amino acid sequence**	**% of Vγ9Jγ2 sequences**	**% of total sequences**
1	ALWEVQLPNYYKKL	24.75	2.56	ASKKTKKL	14.06	1.53
2	ALWEKSKNYYKKL	17.20	1.78	ALWEGENYYKKL	9.58	1.04
3	ALWEVLYKKL	7.02	0.73	ALWEVWRKL	6.03	0.66
4	ALWEVMDYKKL	4.23	0.44	ALWGRNYYKKL	5.07	0.55
5	ALWESEVISNYYKKL	4.10	0.42	ALWEVRFYYKKL	4.73	0.52
6	ALWEVMNKKL	3.92	0.40	ALWEVLLNYYKKL	4.63	0.50
7	ACYYKKL	3.71	0.38	ALWEVPDYYKKL	4.11	0.45
8	ALWEVPEKL	3.34	0.35	ALWEVDYKKL	4.09	0.44
9	ALWEAGNYYKKL	3.12	0.32	ALWETVKKL	4.07	0.44
10	ALWDVRL	2.81	0.29	ALWEDLGKL	3.79	0.41
**Vγ9 JγP**	**CDR3 amino acid sequence**	**% of Vγ9JγP sequences**	**% of total sequences**	**CDR3 amino acid sequence**	**% of Vγ9JγP sequences**	**% of total sequences**
1	ALWEALRLGKKIKV	31.84	3.81	ALWESQELGKKIKV	44.74	30.35
2	ALWEVQELGKKIKV	13.09	1.57	ALWDSYGLGKKIKV	15.55	10.55
3	ALWEQELGKKIKV	13.04	1.56	ALWEVQELGKKIKV	14.28	9.69
4	ALWGQELGKKIKV	5.92	0.71	ALWGQELGKKIKV	9.58	6.50
5	ALWGSELGKKIKV	5.12	0.61	ALWEVQGELGKKIKV	8.13	5.51
6	ALWESQELGKKIKV	4.92	0.59	ALWEQELGKKIKV	3.94	2.67
7	ALQELGKKIKV	4.81	0.58	ALWEIFQELGKKIKV	1.16	0.79
8	ALWEVQGELGKKIKV	4.45	0.53	ALWETQELGKKIKV	1.10	0.75
9	ALWEIFQELGKKIKV	4.28	0.51	ALWEVFELGKKIKV	0.58	0.40
10	ALWDSYGLGKKIKV	2.28	0.27	ALWEVGELGKKIKV	0.36	0.24

## Discussion

In this study, we investigated the γδ TCR repertoires in tumor tissues and matched blood from four patients with IDH1 wild-type GBM. Except in one case, unique tumor-infiltrating GBM-specific γδ T cells used Vγ9Jγ2 sequences whereas blood γδ T cells were dominated by γδ T cells with Vγ9JγP sequences. Based on the TCRγ gene structure, Vγ9Vδ2 T cells with Vγ9Jγ2 sequences should use the Cγ2 segment, whereas canonical γδ T cells with Vγ9JγP sequences found predominantly in the blood are presumed to be Cγ1 users as the JγP segment is much closer to the Cγ1 segment than to the Cγ2 segment. Because γδ T cells developed sequentially from Cγ1-dependent proximal rearrangement to Cγ2-dependent distal rearrangement (32, 60), these two types of Vγ9Vδ2 T cells appear to have developed in different developmental stages.

Upon antigen stimulation, γδ T cells differentiate into two major types of memory T cells: central memory cells, which patrol the blood and secondary lymphoid organs, and effector memory cells, which migrate to peripheral tissues (52). The circulating Vγ9Vδ2 T cells preferentially express inflammatory homing chemokine receptors including CC chemokine receptor (CCR)1, CCR5, CCR8, CXCR3, and C-X-C chemokine receptor type 4 (CXCR4), which are involved in cell migration from the bloodstream to the tumor site, where they display broad and potent antitumoral activity (61–64). Then, activated Vγ9Vδ2 T cells are able to secrete chemokines, such as chemokine ligands (CCL)3, CCL4, chemokine (C-X-C motif) ligand (CXCL)10, and CXCL13, to recruit dendritic cells/macrophages, NK cells, αβ T cells and B cells to the tumor site (53, 65). Vγ9Vδ2 T cells can directly kill tumor cells through the secretion of cytolytic molecules or antibody-dependent cell-mediated cytotoxicity or indirectly prime and modulate immunological functions of other innate and adaptive immune cells leading to the establishment of profound antitumor immunity (53, 66, 67). For example, upon pAg stimulation, Vγ9Vδ2 T cells preferentially differentiate into Th1-like cells with profound interferon-γ and tumor necrosis factor-α responses (49, 54). However, upon stimulation with IPP, Vγ9Vδ2 T cells can also be polarized into Th2-like cells, which are characterized by increased secretion of interleukin (IL)-4 upon stimulation with IL-4 and anti-IL-12 antibodies (54) and Treg-like γδ-T cells with regulatory/immunosuppressive functions in the presence of IL-15 and transforming growth factor-β (55), displaying the functions for promoting tumor development through direct or indirect strategies. To date, it is not known whether these pro-tumor Vγ9Vδ2 T cells have the same clonotypes as antitumor Vγ9Vδ2 T cells. Classical immunotherapeutic approaches to GBM have shown mixed results, and therapies focused on innate lymphocyte activity against GBM have not been rigorously evaluated. The adoption of Vγ9Vδ2 T cells with both canonical and non-canonical Vγ9Vδ2 T cells needs to be evaluated in GBM immunotherapy.

The binding between canonical Vγ9Vδ2 TCR and pAgs presented by BTN3A1 leads to activation of Vγ9Vδ2 T cells (18, 21). Microbe-derived hydroxymethyl-but-2-enyl-pyrophosphate (HMBPP) is ~10,000-fold more potent than cancer cell-derived IPP in the activation of Vγ9Vδ2 T cells (18), but it is not understood how HMBPP triggers stronger signaling in Vγ9Vδ2 T cells than IPP. CDR3γ appeared to be essential for recognition of pyrophosphate-containing metabolites by BTN3A1. In particular, Lys109 in the CDR3γ from the JγP segment was shown to be critical for the binding (68). The CDR3γ sequences from Vγ9Jγ2-Vδ2 T cells showed a shorter length than the CDR3γ sequences from canonical Vγ9Vδ2 T cells with Vγ9JγP sequences, and some clonotypes from Vγ9Vδ2 T cells with Vγ9Jγ2 sequences lacked the critical Lys residue (Supplementary Figure 3). Therefore, further studies are needed to determine whether intratumoral Vγ9Jγ2-Vδ2 T cells recognize the same antigens that bind to the canonical Vγ9Vδ2 T cells with Vγ9JγP sequences with similar affinities.

Collectively, our data showed that the TCR repertoires of intratumoral γδ T cells were clearly distinct from those of blood γδ T cells when comparing TCR repertoires from four patients with GBM. We also identified several GBM-specific Vγ9Jγ2-Vδ2 T cells that were not found in the blood. In-depth investigation of their antigenic specificities and anti- or protumor differentiation potentials through interaction with tumor cells and other stromal cells within GBM-specific TME should be performed to improve our understanding of the roles of γδ T cells in the establishment of the TME and to apply them for the future immunotherapies against GBMs refractory to conventional immunotherapies.

## Author Contributions

D-SK, HL, and TK contributed to the conception and design of the study. ML, CP, JW, D-SK, HL, and TK developed the methodology of the study. ML, CP, JW, JK, IK, D-HN, W-YP, and Y-SK acquired the data, analyzed and interpreted the data. ML, CP, JW, D-SK, HL, and TK wrote the first draft of the manuscript. HL and TK wrote the final form of the manuscript. D-SK, HL, and TK supervised the overall study.

### Conflict of Interest Statement

The authors declare that the research was conducted in the absence of any commercial or financial relationships that could be construed as a potential conflict of interest.

## References

[1] Parney IF Waldron JS Parsa AT. Flow cytometry and *in vitro* analysis of human glioma-associated macrophages. Laboratory investigation. J Neurosurg. (2009) 110:572–82. 10.3171/2008.7.JNS08475 19199469 PMC3064468

[2] Gieryng A Kaminska B. Myeloid-derived suppressor cells in gliomas. Contemp Oncol. (2016) 20:345–51. 10.5114/wo.2016.64592 28373814 PMC5371700

[3] Fecci PE Mitchell DA Whitesides JF Xie W Friedman AH Archer GE. Increased regulatory T-cell fraction amidst a diminished CD4 compartment explains cellular immune defects in patients with malignant glioma. Cancer Res. (2006) 66:3294–302. 10.1158/0008-5472.CAN-05-3773 16540683

[4] Lohr J Ratliff T Huppertz A Ge Y Dictus C Ahmadi R. Effector T-cell infiltration positively impacts survival of glioblastoma patients and is impaired by tumor-derived TGF-beta. Clin Cancer Res. (2011) 17:4296–308. 10.1158/1078-0432.CCR-10-2557 21478334

[5] Rooney MS Shukla SA Wu CJ Getz G Hacohen N. Molecular and genetic properties of tumors associated with local immune cytolytic activity. Cell. (2015) 160:48–61. 10.1016/j.cell.2014.12.033 25594174 PMC4856474

[6] Wang Q Hu B Hu X Kim H Squatrito M Scarpace L. Tumor evolution of glioma-intrinsic gene expression subtypes associates with immunological changes in the microenvironment. Cancer Cell. (2017) 32:42–56 e6. 10.1016/j.ccell.2017.06.003 28697342 PMC5599156

[7] Wang J Cazzato E Ladewig E Frattini V Rosenbloom DI Zairis S. Clonal evolution of glioblastoma under therapy. Nat Genet. (2016) 48:768–76. 10.1038/ng.3590 27270107 PMC5627776

[8] Gabrusiewicz K Ellert-Miklaszewska A Lipko M Sielska M Frankowska M Kaminska B. Characteristics of the alternative phenotype of microglia/macrophages and its modulation in experimental gliomas. PLoS ONE. (2011) 6:e23902. 10.1371/journal.pone.0023902 21901144 PMC3162015

[9] Gieryng A Pszczolkowska D Bocian K Dabrowski M Rajan WD Kloss M. Immune microenvironment of experimental rat C6 gliomas resembles human glioblastomas. Sci Rep. (2017) 7:17556. 10.1038/s41598-017-17752-w 29242629 PMC5730558

[10] Chanmee T Ontong P Konno K Itano N. Tumor-associated macrophages as major players in the tumor microenvironment. Cancers. (2014) 6:1670–90. 10.3390/cancers6031670 25125485 PMC4190561

[11] Piao Y Liang J Holmes L Zurita AJ Henry V Heymach JV. Glioblastoma resistance to anti-VEGF therapy is associated with myeloid cell infiltration, stem cell accumulation, and a mesenchymal phenotype. Neuro Oncol. (2012) 14:1379–92. 10.1093/neuonc/nos158 22965162 PMC3480262

[12] Lim M Xia Y Bettegowda C Weller M. Current state of immunotherapy for glioblastoma. Nat Rev Clin Oncol. (2018) 15:422–42. 10.1038/s41571-018-0003-5 29643471

[13] Fournie JJ Sicard H Poupot M Bezombes C Blanc A Romagne F. What lessons can be learned from gammadelta T cell-based cancer immunotherapy trials? Cell Mol Immunol. (2013) 10:35–41. 10.1038/cmi.2012.39 23241899 PMC4003170

[14] Bryant NL Suarez-Cuervo C Gillespie GY Markert JM Nabors LB Meleth S. Characterization and immunotherapeutic potential of gammadelta T-cells in patients with glioblastoma. Neuro Oncol. (2009) 11:357–67. 10.1215/15228517-2008-111 19211933 PMC2743216

[15] Correia DV Lopes A Silva-Santos B. Tumor cell recognition by gammadelta T lymphocytes: T-cell receptor vs. NK-cell receptors. Oncoimmunology. (2013) 2:e22892. 10.4161/onci.22892 23483102 PMC3583939

[16] Bonneville M O'Brien RL Born WK. Gammadelta T cell effector functions: a blend of innate programming and acquired plasticity. Nat Rev Immunol. (2010) 10:467–78. 10.1038/nri2781 20539306

[17] Vantourout P Hayday A. Six-of-the-best: unique contributions of gammadelta T cells to immunology. Nat Rev Immunol. (2013) 13:88–100. 10.1038/nri3384 23348415 PMC3951794

[18] Gu S Nawrocka W Adams EJ. Sensing of pyrophosphate metabolites by Vgamma9Vdelta2 T Cells. Front Immunol. (2014) 5:688. 10.3389/fimmu.2014.00688 25657647 PMC4303140

[19] Chitadze G Lettau M Luecke S Wang T Janssen O Furst D. NKG2D- and T-cell receptor-dependent lysis of malignant glioma cell lines by human gammadelta T cells: Modulation by temozolomide and A disintegrin and metalloproteases 10 and 17 inhibitors. Oncoimmunology. (2016) 5:e1093276. 10.1080/2162402X.2015.1093276 27141377 PMC4839372

[20] Lefranc MP. The human T-cell rearranging gamma (TRG) genes and the gamma T-cell receptor. Biochimie. (1988) 70:901–8. 3145025 10.1016/0300-9084(88)90231-3

[21] Vavassori S Kumar A Wan GS Ramanjaneyulu GS Cavallari M El Daker S. Butyrophilin 3A1 binds phosphorylated antigens and stimulates human gammadelta T cells. Nat Immunol. (2013) 14:908–16. 10.1038/ni.2665 23872678

[22] Gober HJ Kistowska M Angman L Jeno P Mori L De Libero G. Human T cell receptor gammadelta cells recognize endogenous mevalonate metabolites in tumor cells. J Exp Med. (2003) 197:163–8. 10.1084/jem.20021500 12538656 PMC2193814

[23] Thompson K Dunford JE Ebetino FH Rogers MJ. Identification of a bisphosphonate that inhibits isopentenyl diphosphate isomerase and farnesyl diphosphate synthase. Biochem Biophys Res Commun. (2002) 290:869–73. 10.1006/bbrc.2001.6289 11785983

[24] Kong Y Cao W Xi X Ma C Cui L He W. The NKG2D ligand ULBP4 binds to TCRgamma9/delta2 and induces cytotoxicity to tumor cells through both TCRgammadelta and NKG2D. Blood. (2009) 114:310–7. 10.1182/blood-2008-12-196287 19436053

[25] Dai Y Chen H Mo C Cui L He W. Ectopically expressed human tumor biomarker MutS homologue 2 is a novel endogenous ligand that is recognized by human gammadelta T cells to induce innate anti-tumor/virus immunity. J Biol Chem. (2012) 287:16812–9. 10.1074/jbc.M111.327650 22433851 PMC3351303

[26] Vantourout P Mookerjee-Basu J Rolland C Pont F Martin H Davrinche C. Specific requirements for Vgamma9Vdelta2 T cell stimulation by a natural adenylated phosphoantigen. J Immunol. (2009) 183:3848–57. 10.4049/jimmunol.0901085 19710470 PMC2809082

[27] Zitvogel L Apetoh L Ghiringhelli F Kroemer G. Immunological aspects of cancer chemotherapy. Nat Rev Immunol. (2008) 8:59–73. 10.1038/nri2216 18097448

[28] Robins H. Immunosequencing: applications of immune repertoire deep sequencing. Curr Opin Immunol. (2013) 25:646–52. 10.1016/j.coi.2013.09.017 24140071

[29] Emerson RO Sherwood AM Rieder MJ Guenthoer J Williamson DW Carlson CS. High-throughput sequencing of T-cell receptors reveals a homogeneous repertoire of tumour-infiltrating lymphocytes in ovarian cancer. J Pathol. (2013) 231:433–40. 10.1002/path.4260 24027095 PMC5012191

[30] Chien YH Bonneville M. Gamma delta T cell receptors. Cell Mol Life. (2006) 63:2089–94. 10.1007/s00018-006-6020-z 17003966 PMC11136182

[31] Borst J Wicherink A Van Dongen JJ De Vries E Comans-Bitter WM Wassenaar F. Non-random expression of T cell receptor gamma and delta variable gene segments in functional T lymphocyte clones from human peripheral blood. Eur J Immunol. (1989) 19:1559–68. 10.1002/eji.1830190907 2529123

[32] Tribel F Lefranc MP Hercend T. Further evidence for a sequentially ordered activation of T cell rearranging gamma genes during T lymphocyte differentiation. Eur J Immunol. (1988) 18:789–94. 10.1002/eji.1830180520 2967764

[33] Chen H He X Wang Z Wu D Zhang H Xu C. Identification of human T cell receptor gammadelta-recognized epitopes/proteins via CDR3delta peptide-based immunobiochemical strategy. J Biol Chem. (2008) 283:12528–37. 10.1074/jbc.M708067200 18321859

[34] Xi X Guo Y Chen H Xu C Zhang H Hu H. Antigen specificity of gammadelta T cells depends primarily on the flanking sequences of CDR3delta. J Biol Chem. (2009) 284:27449–55. 10.1074/jbc.M109.011684 19666468 PMC2785674

[35] Dobin A Davis CA Schlesinger F Drenkow J Zaleski C Jha S. STAR: ultrafast universal RNA-seq aligner. Bioinformatics. (2013) 29:15–21. 10.1093/bioinformatics/bts635 23104886 PMC3530905

[36] Li B Dewey CN RSEM: accurate transcript quantification from RNA-Seq data with or without a reference genome. BMC Bioinformatics. (2011) 12:323 10.1186/1471-2105-12-323 21816040 PMC3163565

[37] Newman AM Liu CL Green MR Gentles AJ Feng W Xu Y. Robust enumeration of cell subsets from tissue expression profiles. Nat Methods. (2015) 12:453–7. 10.1038/nmeth.3337 25822800 PMC4739640

[38] Azizi E Carr AJ Plitas G Cornish AE Konopacki C Prabhakaran S. Single-cell map of diverse immune phenotypes in the breast tumor microenvironment. Cell. (2018) 174:1293–308 e36. 10.1016/j.cell.2018.05.060 29961579 PMC6348010

[39] Chung W Eum HH Lee HO Lee KM Lee HB Kim KT. Single-cell RNA-seq enables comprehensive tumour and immune cell profiling in primary breast cancer. Nat Commun. (2017) 8:15081. 10.1038/ncomms15081 28474673 PMC5424158

[40] De Simone M Arrigoni A Rossetti G Gruarin P Ranzani V Politano C Transcriptional landscape of human tissue lymphocytes unveils uniqueness of tumor-infiltrating T regulatory cells. Immunity. (2016) 45:1135–47. 10.1016/j.immuni.2016.10.021 27851914 PMC5119953

[41] Finotello F Trajanoski Z. Quantifying tumor-infiltrating immune cells from transcriptomics data. Cancer Immunol Immunother. (2018) 67:1031–40. 10.1007/s00262-018-2150-z 29541787 PMC6006237

[42] Hanzelmann S Castelo R Guinney J. GSVA: gene set variation analysis for microarray and RNA-seq data. BMC Bioinformatics. (2013) 14:7. 10.1186/1471-2105-14-7 23323831 PMC3618321

[43] GR Warnes BB L Bonebakker R Gentleman WHA Liaw T Lumley gplots: Various R Programming Tools for Plotting Data. R package version 2.12. 1. 2013. Google Scholar (2014).

[44] Giudicelli V Duroux P Ginestoux C Folch G Jabado-Michaloud J Chaume D. IMGT/LIGM-DB, the IMGT comprehensive database of immunoglobulin and T cell receptor nucleotide sequences. Nucleic Acids Res. (2006) 34:D781–4. 10.1093/nar/gkj088 16381979 PMC1347451

[45] Li B Li T Pignon JC Wang B Wang J Shukla SA. Landscape of tumor-infiltrating T cell repertoire of human cancers. Nat Genet. (2016) 48:725–32. 10.1038/ng.3581 27240091 PMC5298896

[46] Louis DN Perry A Reifenberger G von Deimling A Figarella-Branger D Cavenee WK. The 2016 World Health Organization classification of tumors of the central nervous system: a summary. Acta Neuropathol. (2016) 131:803–20. 10.1007/s00401-016-1545-1 27157931

[47] Stupp R Mason WP van den Bent MJ Weller M Fisher B Taphoorn MJ. Radiotherapy plus concomitant and adjuvant temozolomide for glioblastoma. N Engl J Med. (2005) 352:987–96. 10.1056/NEJMoa043330 15758009

[48] Tosolini M Pont F Poupot M Vergez F Nicolau-Travers ML Vermijlen D. Assessment of tumor-infiltrating TCRVgamma9Vdelta2 gammadelta lymphocyte abundance by deconvolution of human cancers microarrays. Oncoimmunology. (2017) 6:e1284723. 10.1080/2162402X.2017.1284723 28405516 PMC5384348

[49] Dunne MR Mangan BA Madrigal-Estebas L Doherty DG. Preferential Th1 cytokine profile of phosphoantigen-stimulated human Vgamma9Vdelta2 T cells. Mediators Inflamm. (2010) 2010:704941. 10.1155/2010/704941 21403900 PMC3043297

[50] Caccamo N La Mendola C Orlando V Meraviglia S Todaro M Stassi G. Differentiation, phenotype, and function of interleukin-17-producing human Vgamma9Vdelta2 T cells. Blood. (2011) 118:129–38. 10.1182/blood-2011-01-331298 21505189

[51] Mao Y Yin S Zhang J Hu Y Huang B Cui L. A new effect of IL-4 on human gammadelta T cells: promoting regulatory Vdelta1 T cells via IL-10 production and inhibiting function of Vdelta2 T cells. Cell Mol Immunol. (2016) 13:217–28. 10.1038/cmi.2015.07 25942601 PMC4786628

[52] Caccamo N Todaro M Sireci G Meraviglia S Stassi G Dieli F. Mechanisms underlying lineage commitment and plasticity of human gammadelta T cells. Cell Mol Immunol. (2013) 10:30–4. 10.1038/cmi.2012.42 23085943 PMC4003171

[53] Tyler CJ Doherty DG Moser B Eberl M. Human Vgamma9/Vdelta2 T cells: Innate adaptors of the immune system. Cell Immunol. (2015) 296:10–21. 10.1016/j.cellimm.2015.01.008 25659480

[54] Wesch D Glatzel A Kabelitz D Differentiation of resting human peripheral blood gamma delta T cells toward Th1- or Th2-phenotype. Cell Immunol. (2001) 212:110–7. 10.1006/cimm.2001.1850 11748927

[55] Casetti R Agrati C Wallace M Sacchi A Martini F Martino A. Cutting edge: TGF-beta1 and IL-15 Induce FOXP3+ gammadelta regulatory T cells in the presence of antigen stimulation. J Immunol. (2009) 183:3574–7. 10.4049/jimmunol.0901334 19710458

[56] Wu YL Ding YP Tanaka Y Shen LW Wei CH Minato N. gammadelta T cells and their potential for immunotherapy. Int J Biol Sci. (2014) 10:119–35. 10.7150/ijbs.7823 24520210 PMC3920167

[57] De Rosa SC Andrus JP Perfetto SP Mantovani JJ Herzenberg LA Herzenberg LA Ontogeny of gamma delta T cells in humans. J Immunol. (2004) 172:1637-45. 10.4049/jimmunol.172.3.1637 14734745

[58] Sherwood AM Desmarais C Livingston RJ Andriesen J Haussler M Carlson CS. Deep sequencing of the human TCRgamma and TCRbeta repertoires suggests that TCRbeta rearranges after alphabeta and gammadelta T cell commitment. Sci Transl Med. (2011) 3:90ra61. 10.1126/scitranslmed.3002536 21734177 PMC4179204

[59] Nazarov VI Pogorelyy MV Komech EA Zvyagin IV Bolotin DA Shugay M. tcR: an R package for T cell receptor repertoire advanced data analysis. BMC Bioinformatics. (2015) 16:175. 10.1186/s12859-015-0613-1 26017500 PMC4445501

[60] Krangel MS Yssel H Brocklehurst C Spits H. A distinct wave of human T cell receptor gamma/delta lymphocytes in the early fetal thymus: evidence for controlled gene rearrangement and cytokine production. J Exp Med. (1990) 172:847–59. 2167345 10.1084/jem.172.3.847 PMC2188534

[61] Bouet-Toussaint F Cabillic F Toutirais O Le Gallo M Thomas de la Pintiere C Daniel P. Vgamma9Vdelta2 T cell-mediated recognition of human solid tumors. Potential for immunotherapy of hepatocellular and colorectal carcinomas. Cancer Immunol Immunother. (2008) 57:531–9. 10.1007/s00262-007-0391-3 17764010 PMC11030195

[62] Cipriani B Borsellino G Poccia F Placido R Tramonti D Bach S. Activation of C-C beta-chemokines in human peripheral blood gammadelta T cells by isopentenyl pyrophosphate and regulation by cytokines. Blood. (2000) 95:39–47. 10607682

[63] Glatzel A Wesch D Schiemann F Brandt E Janssen O Kabelitz D. Patterns of chemokine receptor expression on peripheral blood gamma delta T lymphocytes: strong expression of CCR5 is a selective feature of V delta 2/V gamma 9 gamma delta T cells. J Immunol. (2002) 168:4920–9. 10.4049/jimmunol.168.10.4920 11994442

[64] Hannani D Ma Y Yamazaki T Dechanet-Merville J Kroemer G Zitvogel L. Harnessing gammadelta T cells in anticancer immunotherapy. Trends Immunol. (2012) 33:199–206. 10.1016/j.it.2012.01.006 22364810

[65] Conti L Casetti R Cardone M Varano B Martino A Belardelli F. Reciprocal activating interaction between dendritic cells and pamidronate-stimulated gammadelta T cells: role of CD86 and inflammatory cytokines. J Immunol. (2005) 174:252–60. 10.4049/jimmunol.174.1.252 15611247

[66] Ferreira LM. Gammadelta T cells: innately adaptive immune cells? Int Rev Immunol. (2013) 32:223–48. 10.3109/08830185.2013.783831 23617235

[67] Tokuyama H Hagi T Mattarollo SR Morley J Wang Q So HF. V gamma 9 V delta 2 T cell cytotoxicity against tumor cells is enhanced by monoclonal antibody drugs–rituximab and trastuzumab. Int J Cancer. (2008) 122:2526–34. 10.1002/ijc.23365 18307255

[68] Wang H Fang Z Morita CT. Vgamma2Vdelta2 T Cell Receptor recognition of prenyl pyrophosphates is dependent on all CDRs. J Immunol. (2010) 184:6209–22. 10.4049/jimmunol.1000231 20483784 PMC3069129

