# Supplementary material for: Preferential Infiltration of Unique Vγ9Jγ2-Vδ2 T Cells Into Glioblastoma Multiforme
Source: Front Immunol. 2019 Mar 22;10:555. doi: 10.3389/fimmu.2019.00555 (PMC6440384; doi:10.3389/fimmu.2019.00555)
Supplement: Supplementary file 1 [file Data_Sheet_1.PDF]

Supplementary figure 1

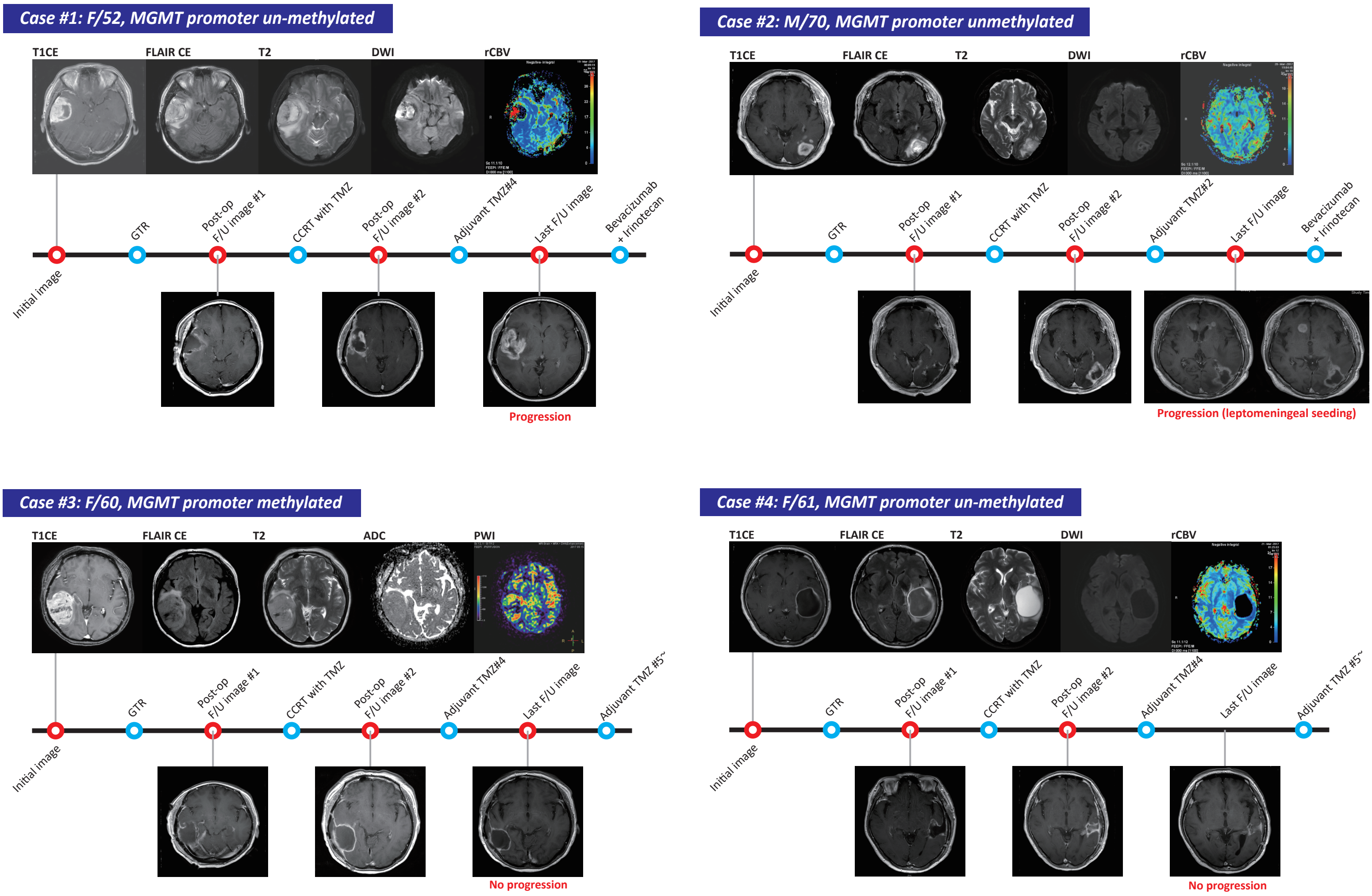

Supplementary figure 1. Clinical course of four GBM patients.

MR images for the geographic sampling panel cases. T1CE, T1-weighted contrast-enhanced MRI; FLAIR CE, fluid attenuation inversion recovery contrast-enhanced magnetic resonance imaging (MRI), which allows for more sensitive detection of small hyperintense lesions than T1CE; DWI, diffusion-weighted MRI, which allows the mapping of the diffusion process of molecules, mainly water, in biological tissues, in vivo and noninvasively, revealing microscopic details about tissue architecture, either normal or in a diseased state; rCBV, relative cerebral blood volume usually obtained by dynamic susceptibility contrast MRI using normal -appearing white matter as a reference region; ADC, apparent diffusion coefficient, which is a measure of the magnitude of diffusion (of water molecules) within tissue and is commonly clinically calculated using MRI with diffusion weighted imaging; GTR, gross total resection; CCRT, concurrent chemo-radiation; TMZ, temozolomide; F/U, follow up. Lt.: left; Rt.: right; MGMT: O6-methylguanine-DNA-methyltransferase.

Supplementary Figure 2

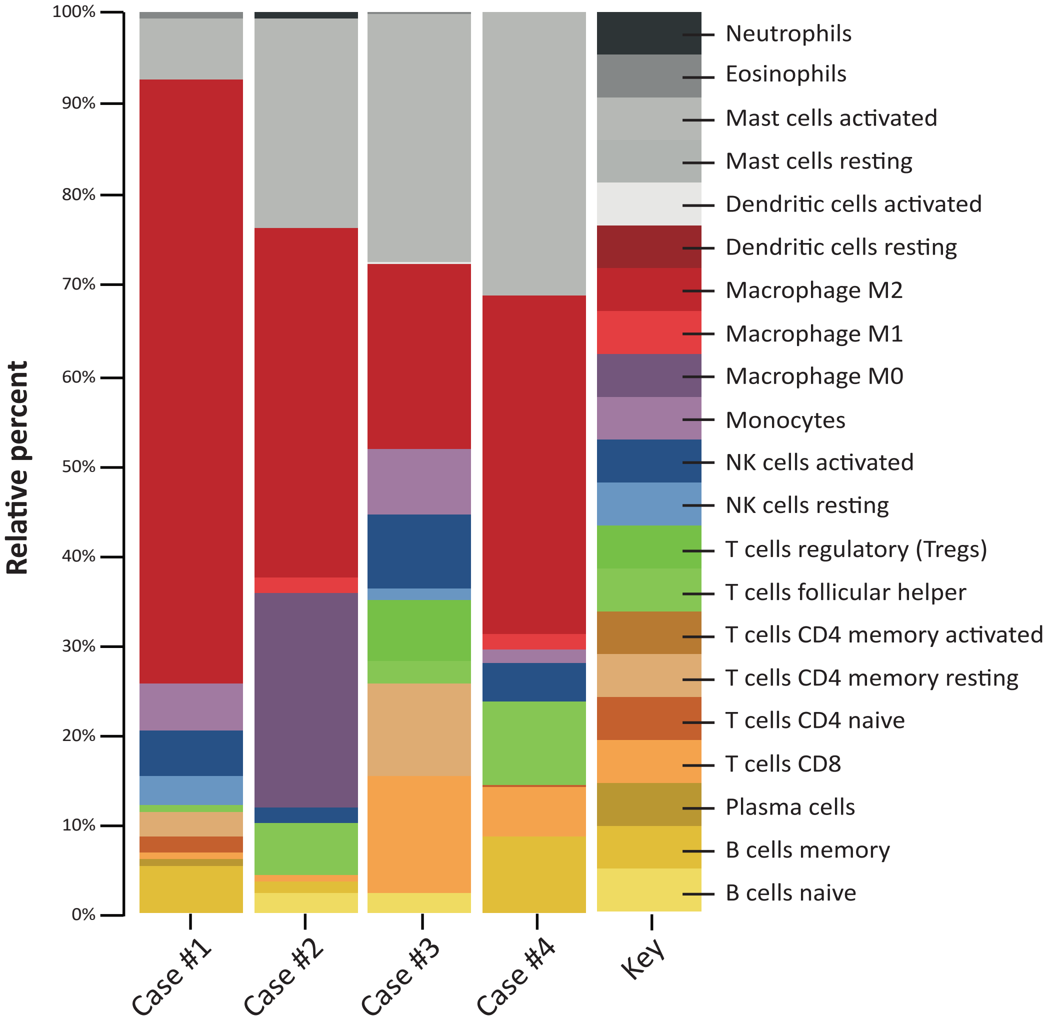

Supplementary figure 2. Immune cell proportions in four case of GBM tumor tissues

Relative levels (percentages) of 21 immune cell subtypes are quantified from GBM RNA-seq using CIBERSORT analysis and represented in a stacked plots.

Supplement figure 3

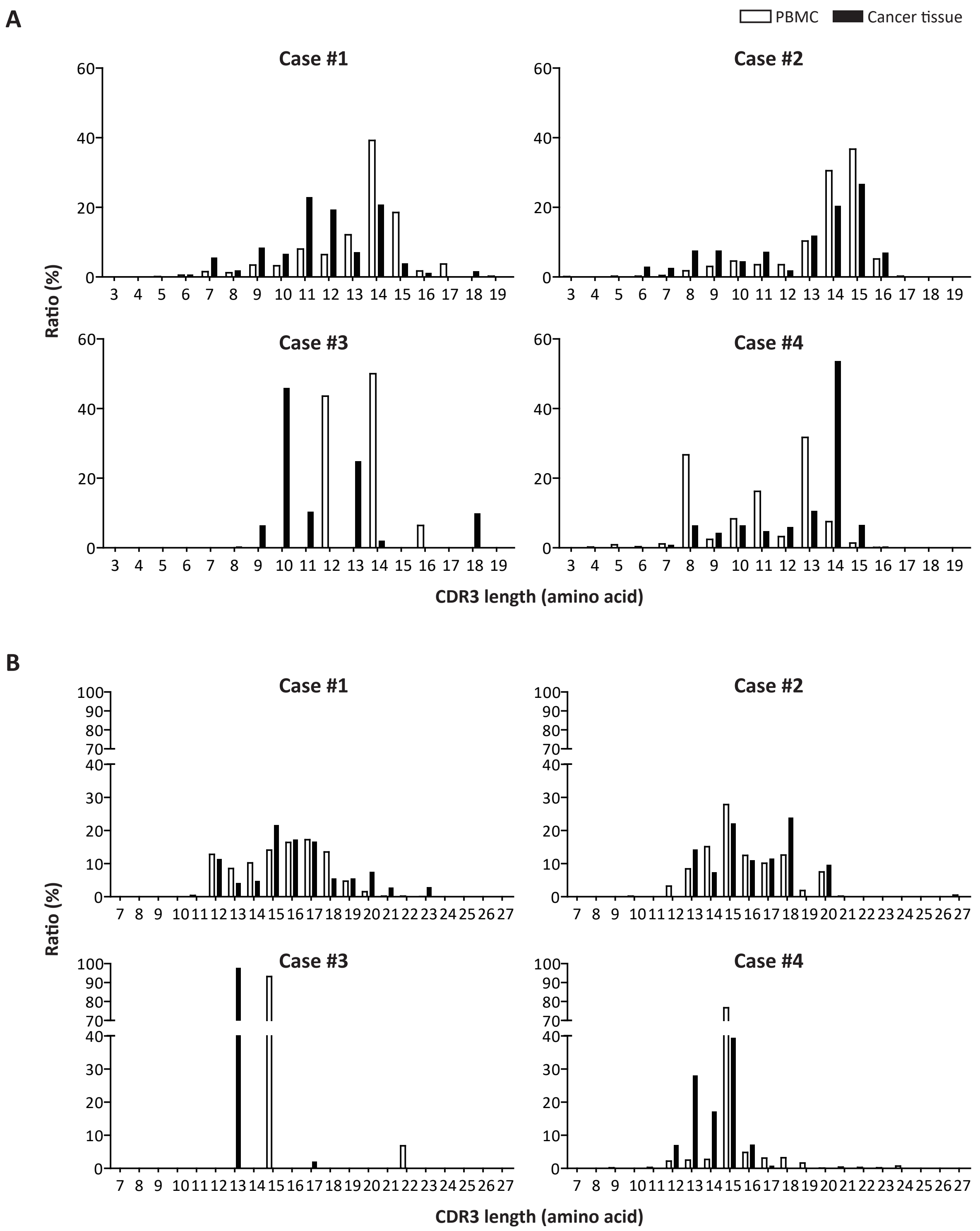

Supplementary figure 3. CDR3 length distributions of γδTCR from blood and GBM cancer tissue

The CDR3 length distributions of TCRγ(A) or TCRδ (B) in PBMCs (white) or cancer tissues (black) for each patient were shown.

Supplementary table 1. List of immune gene signatures

Cell type	Signature genes
γδ T	TRGC2, TRD, CD3D, CD3E, CD28, KLRK1, KLRC1, KLRC2, KLRC3, KLRC4, KLRD1, CD160, NKG7, GZMB, FASLG, IL18RAP, CCL3, CCL4, CCL5, XCL1, XCL2
Th1	CD3E, CD4, TBX21, IFNG, TNF, IL2, IL12RB1, IL12RB2, STAT1
Th2	CD3E, CD4, GATA3, IL4, IL5, IL13, CCL13, CXCL12, TNF
Th17	CD3E, CD4, RORA, RORG, IL17A, IL17F, IL21, STAT3, BATF
Treg	CD3E, CD4, TGFB1, FOXP3, IL2RA, IL10, CTLA4, MAF
CTL	CD3E, CD4, FASL, PRF1, GZMA, GZMB, GZMK, IFNG
Exhausted T	CD3E, CD4, PDCD1, LAG3, TIM3, BTLA, CTLA4, FAS
M1 Macrophage	IL12, IL23, IL12, TNF, IL6, CD86, MHCII, IL1B, MARCO, iNOS, IL12, CD64, CD80, CXCR10, IL23, CXCL9, CXCL10, CXCL11, CD86, IL1A, IL1B, IL6, TNFa, MHCII, CCL5, IRF5, IRF1, CD40, IDO1, KYNU, CCR7, CD45, CD68, CD115, HLA-DR, CD205, CD14
M2 Macrophage	ARG1, ARG2, IL10, CD32, CD163, CD23, CD200R1, PD-L2, PDL1, MARCO, CSF1R, CD206, IL1RN, IL1R2, IL4R, CCL4, CCL13, CCL20, CCL17, CCL18, CCL22, CCL24, LYVE1, VEGFA, VEGFB, VEGFC, VEGFD, EGF, CTSA, CTSB, CSTC, CTSD, TGFB1, TGFB2, TGFB3, MMP14, MMP19, MMP9, CLEC7A, WNT7B, FASL, TNFSF12, TNFSF8, CD276, VTCN1, MSR1, FN1, IRF4, CD45, CD68, CD115, HLA-DR, CD205, CD14

Th1: T helper type 1; Th2: T helper type 2; Th17: T helper type 17; Treg: regulatory T cells; CTL: Cytotoxic T lineage
